# Butterworth Filtering at 500 Hz Optimizes PPG-Based Heart Rate Variability Analysis for Wearable Devices: A Comparative Study

**DOI:** 10.3390/s25227091

**Published:** 2025-11-20

**Authors:** Nagima Abdrasulova, Milana Aleksanyan, Min Ju Kim, Jae Mok Ahn

**Affiliations:** Division of Software, Hallym University, 1 Hallymdaehak-gil, Chuncheon 24252, Gangwon-do, Republic of Korea

**Keywords:** heart rate variability, photoplethysmography, IIR filters, Butterworth filter, elliptic filter, signal processing

## Abstract

**Highlights:**

**What are the main findings?**

- Butterworth filters at ≥500 Hz demonstrated excellent concordance with ECG (CCC ≥ 0.94) and statistical equivalence across frequency-domain HRV parameters.
- Elliptic filters at 250 Hz showed lower concordance (CCC ≈ 0.64) and systematic bias due to ripple and nonlinear phase effects.

**What is the implication of the main finding?**

- Butterworth filtering at 500 Hz offers practical balance between accuracy and resource efficiency for real-time PPG-based HRV monitoring in wearable devices.
- Establishing ≥500 Hz Butterworth filtering as a standard ensures reliable interchangeability of PPG- and ECG-derived HRV for clinical and research applications.

**Abstract:**

Photoplethysmography (PPG)-based heart rate variability (HRV) offers a cost-effective alternative to electrocardiography (ECG) for autonomic monitoring in wearable devices. We optimized signal processing on a 16-bit microcontroller by comparing 4th-order equivalent Butterworth and Elliptic IIR bandpass filters (0.8–20 Hz, zero-phase) at 1000, 500, and 250 Hz. Paired PPG–ECG recordings from 10 healthy adults were analyzed for ln HF, ln LF, and ln VLF using Lin’s concordance correlation coefficient (CCC), ±5% equivalence testing (TOST), and Passing–Bablok regression (PBR). Butterworth at 500 Hz preserved near-identity with ECG standard (CCC ≥0.94; TOST met equivalence; PBR slopes/intercepts: ln HF = 0.97x + 0.10, ln LF = 1.02x − 0.07, ln VLF = 1.01x − 0.03), while halving computational load. In contrast, Elliptic at 250 Hz degraded concordance (CCC ≈ 0.64) and failed equivalence, with greater bias from nonlinear phase and ripple-induced distortion. Elliptic performance improved at higher sampling but offered no benefit over Butterworth. These results support zero-phase Butterworth filtering at ≥500 Hz as the optimal balance of fidelity, robustness, and efficiency, enabling reliable PPG-HRV monitoring on low-power devices. As a pilot investigation (*n* = 10), this study establishes preliminary design parameters and optimal configurations to guide subsequent large-scale clinical validation.

## 1. Introduction

Heart rate variability (HRV) constitutes a recognized non-invasive metric of autonomic nervous system (ANS) function, widely adopted to assess cardiovascular health, stress, and disease progression in both clinical and ambulatory settings [1,2]. While electrocardiography (ECG) remains the traditional reference for HRV analysis, photoplethysmography (PPG) has emerged as an attractive alternative due to its integration into wearable devices, cost-effectiveness, and convenience [3,4]. By detecting pulsatile blood volume changes through optical sensors, PPG enables continuous monitoring without electrodes [5]. However, PPG signals exhibit high susceptibility to motion-induced artifacts, baseline drift, and waveform variability, which complicate peak detection and frequency-domain analysis [6,7].

Robust signal preprocessing is essential to ensure reliable PPG-derived HRV. Bandpass filtering is particularly critical, as the filter design directly influences spectral fidelity, artifact resistance, and computational feasibility in embedded systems. Butterworth filters provide a maximally flat passband (<0.1 dB ripple), preserving waveform integrity, whereas Elliptic filters achieve sharper transitions at the expense of nonlinear phase response and up to 1 dB ripple, which may distort HRV indices [8,9,10]. Furthermore, although ECG guidelines suggest 250–500 Hz sampling for accurate R-peak detection, PPG often requires higher sampling frequencies to avoid aliasing and preserve high-frequency (HF) spectral components [11,12].

Recent studies have investigated optimal sampling frequencies for PPG-based HRV analysis. Béres et al. [13] demonstrated that sampling frequencies as low as 20–50 Hz can be sufficient for time-domain HRV parameters (SDNN, RMSSD) when combined with interpolation techniques. However, their study did not systematically evaluate the effects of filter characteristics or focus on frequency-domain parameters, which are critical for autonomic function assessment. Furthermore, real-time wearable applications often require processing without post hoc interpolation to minimize computational overhead. The present study extends this line of research by comparing filter types (Butterworth vs. Elliptic) across multiple sampling frequencies (1000, 500, 250 Hz) with direct ECG validation, focusing on frequency-domain HRV indices.

Our previous work [14,15] compared Butterworth and Elliptic filters for PPG-based HRV analysis, demonstrating that Butterworth filters yielded more stable NN interval extraction. However, that study did not systematically evaluate the effects of sampling frequency on filter performance, nor did it employ direct PPG-ECG comparison with multiple statistical validation methods. These results highlighted the need for a comprehensive comparison of filtering strategies across multiple sampling rates (1000, 500, 250 Hz), incorporating Frequency–response and pole–zero analyses, together with rigorous statistical validation against simultaneously recorded ECG-derived references.

Despite the growing adoption of PPG-based wearables, there remains a lack of evidence-based guidelines for optimal filter-sampling configurations that balance measurement accuracy with computational efficiency. While higher sampling rates (≥1000 Hz) ensure signal fidelity, they impose significant computational burden on battery-constrained devices. Conversely, aggressive downsampling may compromise high-frequency HRV components. Establishing the minimum sampling frequency that preserves clinical-grade accuracy is therefore critical for next-generation wearable HRV monitors.

The present study aims to identify the optimal filter–sampling configuration for PPG-based HRV analysis in resource-constrained wearable platforms. Specifically, we compare zero-phase Butterworth and Elliptic infinite impulse response (IIR) bandpass filters (0.8–20 Hz) at 1000, 500, and 250 Hz, and evaluate their interchangeability with ECG-derived HRV indices using concordance correlation coefficient (CCC), two one-sided equivalence testing (TOST), Passing–Bablok regression, and Bland–Altman analysis [16,17,18,19]. By jointly addressing accuracy and computational feasibility, this work provides design guidance for low-power wearable devices and establishes a robust foundation for future machine learning–based artifact suppression and broader clinical validation [20].

This pilot investigation with 10 healthy participants represents a critical first step in establishing evidence-based design parameters for next-generation wearable HRV monitors. While the modest sample size limits generalizability, our systematic approach—combining rigorous statistical validation, computational efficiency analysis, and direct PPG-ECG comparison—provides essential preliminary guidance for optimizing filter-sampling configurations before undertaking large-scale clinical trials or commercial product development. The findings presented here are intended to inform the design of subsequent multicenter validation studies involving diverse patient populations and real-world ambulatory conditions.

## 2. Materials and Methods

### 2.1. IIR Filter Design and Implementation

The IIR filter design followed standard procedures detailed in [8,14]. Briefly, the recursive IIR filter can be expressed as:

(1) $$y\left[n\right]=\sum_{k=0}^{M}b_{k}x\left[n-k\right]-\sum_{k=1}^{N}a_{k}y\left[n-k\right]$$

where the notations *x*[n] and *y*[n] represent the input and output sequences, respectively, and a_k_, b_k_ are the filter coefficients of the Nth-order and Mth-order filter. For complete derivation, see our previous work [14].

In this study, we designed and applied 2nd-order IIR bandpass filters with cutoff frequencies of 0.8 Hz (high-pass) and 20 Hz (low-pass). To eliminate phase distortion, we implemented zero-phase forward-backward filtering using the scipy.signal.filtfilt function. This approach applies the filter twice—once forward and once backward—effectively producing a 4th-order equivalent response with zero phase shift [9]. The Butterworth design provides a maximally flat passband (<0.1 dB ripple), whereas the Elliptic filter achieves sharper transitions and stronger stopband attenuation, but introduces nonlinear phase and passband ripple of up to 1 dB [21]. These properties directly influence temporal accuracy in HRV analysis, since group delay variability can bias beat-to-beat interval detection and spectral power estimation [20]. Recent studies have confirmed that filtering strategies significantly impact PPG-derived HRV. In particular, Mejía-Mejía et al. demonstrated that filter selection alters concordance between PRV and ECG-HRV, highlighting the importance of minimizing timing distortion and phase irregularities in preprocessing [22]. Based on these considerations, both Butterworth and Elliptic designs were evaluated at sampling frequencies of 1000, 500, and 250 Hz, with coefficients computed in Python 3.10.9 using the scipy 1.10.0 package. The final coefficients are summarized in Table 1.

### 2.2. Frequency Response Analysis

Frequency-domain characterization was conducted to compare the Butterworth and Elliptic bandpass filters (0.8–20 Hz) at sampling frequencies of 1000, 500, and 250 Hz. Both filters exhibited a low-frequency transition width of approximately 0.65 Hz, while the Elliptic design achieved narrower high-frequency transitions due to the presence of transmission zeros. The group delay, which quantifies the time-domain latency introduced by the filter, was defined as:

(2) $$\tau_{g}\left(\omega \right)=-\frac{d\phi \left(\omega \right)}{d\omega }$$

where $\phi \left(\omega \right)$ denotes the filter’s phase response. Group delay analysis is critical in biomedical signal processing because latency variations in the passband can distort beat-to-beat interval estimation, thereby affecting HRV accuracy [23,24]. Ideally, a filter should exhibit constant group delay across the passband to ensure uniform time-domain behavior for all frequency components within the signal of interest. Butterworth filters are known for their relatively flat group delay characteristics, which contribute to minimal waveform distortion and predictable peak timing in PPG signals. In contrast, Elliptic filters—despite their superior frequency selectivity—exhibit frequency-dependent group delay variations, particularly near passband edges, which can lead to temporal inconsistencies in peak detection and inter-beat interval (IBI) measurement [8,21].

**Figure 1:**
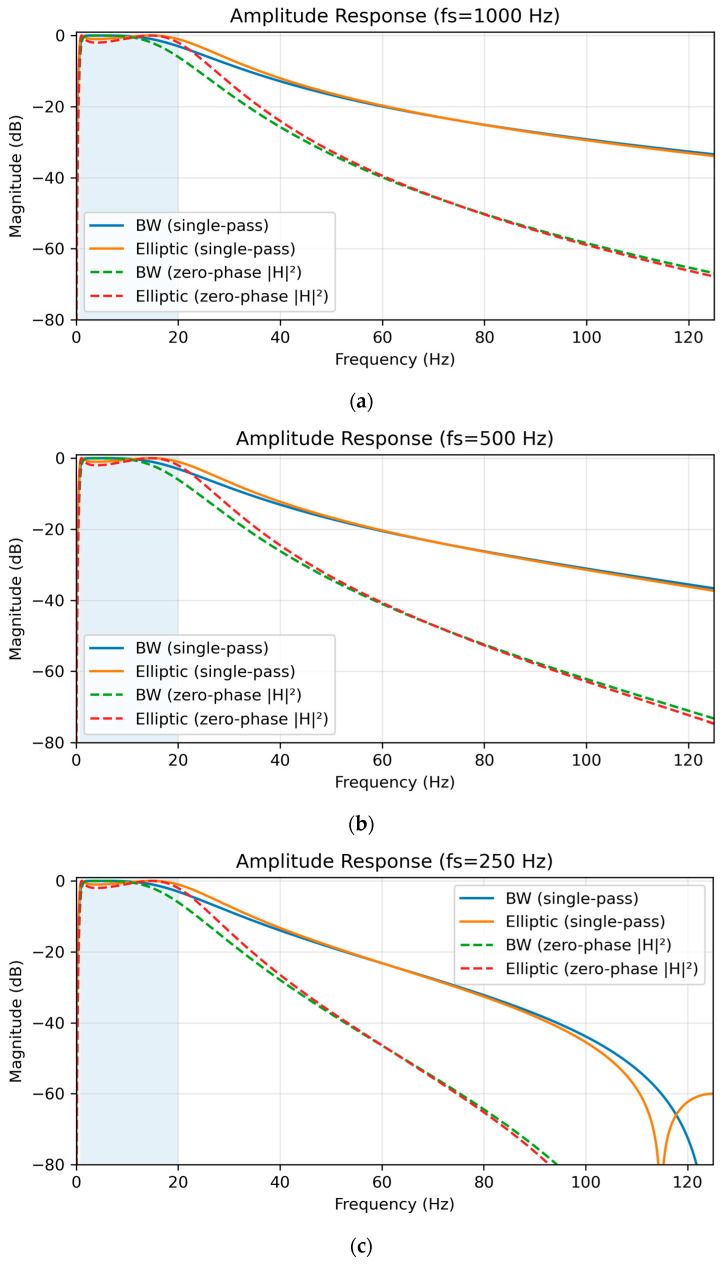
Amplitude response of Butterworth and Elliptic bandpass filters (0.8–20 Hz, 2nd-order) under single-pass (solid lines) and zero-phase forward-backward (dashed lines) implementations, with the passband shaded. (**a**) At 1000 Hz, Butterworth shows a flat passband (transition widths: 0.65 Hz low, 84.35 Hz high), while Elliptic has 1 dB ripple and sharper roll-off (82.80 Hz high). (**b**) At 500 Hz, Butterworth (0.65 Hz low, 75.36 Hz high) and Elliptic (74.18 Hz high) maintain spectral fidelity. (**c**) At 250 Hz, Butterworth (0.65 Hz low, 55.34 Hz high) and Elliptic (54.74 Hz high) show reduced high-frequency transitions due to lower Nyquist frequency.

Amplitude responses for each sampling condition are shown in Figure 1. Single-pass causal filtering (solid lines) was contrasted with zero-phase forward–backward filtering (dashed lines). The latter squared the magnitude response (|H|^2^), effectively doubling the filter order (from 2nd to 4th) and yielding steeper attenuation (~24 dB/octave) without phase distortion [9]. At 1000 Hz, the Butterworth filter maintained a flat passband (ripple ≈ 0 dB) with transition widths of 0.65 Hz (low) and 84.35 Hz (high), whereas the Elliptic filter demonstrated 1 dB ripple and sharper roll-off (82.80 Hz). At 500 Hz, both filters preserved spectral fidelity, though Elliptic again displayed stronger selectivity with ±80° phase variation and group delay spread ranging from 6 to 145 samples. At 250 Hz, Butterworth transition widths were 0.65 Hz (low) and 55.34 Hz (high), while Elliptic achieved 54.74 Hz (high) but introduced variability in group delay (3–72 samples, mean 37.5 ± 11.0), which may exacerbate artifact sensitivity in noisy environments. Table 2 summarizes the main Frequency–response parameters, including transition widths, passband ripple, group-delay spread, and phase variation.

**Figure 2:**
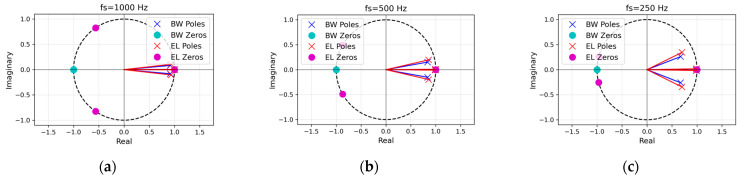
Pole–zero diagrams of the 2nd-order digital Butterworth (BW, blue) and elliptic (EL, red) bandpass filters (0.8–20 Hz) at three sampling rates: (**a**) $f_{s}\;$ = 1000 Hz, (**b**) $f_{s}\;$= 500 Hz, (**c**) $f_{s}\;$= 250 Hz. The poles are marked with × and transmission zeros with ○, enclosed by a dashed unit circle. Both designs place zeros at *z* = +1 and *z* = −1 (suppressing DC and Nyquist), while the elliptic filter additionally introduces two transmission zeros positioned on the unit circle in the left half-plane, producing sharper transition bands. All poles lie inside the unit circle (stable); pole radii decrease slightly as $f_{s}$ decreases because with the 0.8–20 Hz passband fixed, the natural decrease in pole radii results from pole readjustment to maintain filter stability.

In addition to magnitude analysis, pole–zero (PZ) plots (Figure 2) were generated to verify filter stability and interpret spectral behavior. All poles remained inside the unit circle (∣z∣ < 1), ensuring stability. Butterworth filters showed zeros at *z* = ±1, yielding DC and Nyquist suppression with smooth pole distributions, resulting in a flat passband and temporal stability. In contrast, Elliptic filters introduced additional transmission zeros near the stopbands, enabling sharper transitions and stronger attenuation at the cost of ripple and nonlinear phase [21]. As the sampling frequency decreased, normalized pole and zero positions shifted toward the Nyquist limit, extending impulse response durations, particularly for Elliptic filters. This geometric interpretation highlights Butterworth’s suitability for maintaining beat-to-beat timing precision, while Elliptic prioritizes frequency selectivity and baseline wander suppression. This PZ analysis is critical for PPG-HRV and ECG-HRV concordance, as it addresses two key factors: (1) temporal precision of beat intervals which is crucial for HRV analysis, enhanced by Butterworth’s flat passband and linear phase, reducing peak-picking bias relative to elliptic ripple, particularly at low $f_{s}$; and (2) artifact rejection, improved by Elliptic’s deeper stopband zeros for suppressing baseline wander and motion noise [23]. Optimal filter configurations—stable poles with moderate radii, balanced stopband zeros, and zero-phase application—were identified via PZ geometry. Consequently, the geometrical difference in pole and zero locations demonstrates the Butterworth filter’s emphasis on temporal stability, while the Elliptic filter prioritizes frequency selectivity.

### 2.3. Filter Pole-Zero Geometry Optimization for PPG-Based HRV Processing

The influence of pole geometry on the bandpass characteristics was investigated using a 2-pole Butterworth prototype, mapped into the digital domain through the bilinear transform [10]:

(3) $$z=\frac{1+\frac{T_{s}}{2}s}{1-\frac{T_{s}}{2}s}$$

where $T_{s}=1/f_{s}$ is the sampling period, and cutoff frequencies were set to 0.8 Hz and 20 Hz. At sampling rates of 1000, 500, and 250 Hz, the corresponding discrete pole angles were approximately 5°, 10°, and 26°, with pole radius r ≈ 0.95, ensuring passband flatness and system stability. Lower sampling frequencies shortened the step-response settling time under zero-phase implementation (from ~90 ms to ~35 ms) and improved rejection of 60 Hz interference (attenuation from –19.9 dB to –23.2 dB in single-pass mode). The 500 Hz condition (θ ≈ 10°) provided the best balance between transient duration, stopband suppression, and numerical stability, and was therefore selected for subsequent experiments, supplemented by an adaptive 60 Hz IIR notch filter [25].

### 2.4. Signal Processing and Peak Detection

To remove phase distortion, zero-phase filtering was achieved using forward–backward processing. Applying this method to 2nd-order Butterworth and Elliptic bandpass filters effectively yielded 4th-order characteristics. The transfer function is expressed as [26]:

(4) $$H_{\mathrm{zp}}\left(z\right)=H\left(z\right)H\left(z^{-1}\right)$$

with the resulting magnitude and phase given by:

(5) $${\left|H_{\mathrm{zp}}\left(e^{j\omega }\right)\right|}^{2}={\left|H\left(e^{j\omega }\right)\right|}^{2}$$

(6) $$\phi_{\mathrm{zp}}\left(\omega \right)=0$$

This method preserved the filter’s desirable characteristics, such as the Butterworth’s maximally flat passband (ripple < 0.1 dB), while simultaneously enhancing stopband attenuation by effectively doubling the filter order without introducing latency. Forward–backward filtering has been widely used in biomedical signal processing for HRV and ECG analysis, as it combines high-order attenuation with phase preservation [9,27].

Following the filtering process, peak detection was performed on the filtered signals, which is standard practice in PPG signal analysis, as filtering reduces baseline wander and noise, thereby improving the reliability of peak detection. The peak detection parameters were consistent across all three sampling frequencies (1000, 500, 250 Hz), as the physiological constraints (minimum RR interval of 300 ms) remain independent of the sampling rate. For the ECG signals, R-peaks were detected using the well-established Pan-Tompkins algorithm. To guarantee the accuracy of the final NN/PP interval data, all automatically detected peaks were visually inspected and manually corrected where necessary.

### 2.5. Study Participants and Sample Size Determination

This study adhered to the principles of the Declaration of Helsinki and obtained ethical approval from the Institutional Review Board of Hallym University. Written informed consent was obtained from all participants prior to their enrollment in the study.

The sample size was determined based on Lin’s established methodology for concordance correlation coefficient (CCC) analysis [16]. Lin [16] establishes that CCC can be legitimately calculated on as few as 10 observations, representing the minimum statistically valid sample size for agreement studies. While formal power analysis for detecting a target CCC of 0.90 versus a null hypothesis of 0.80 (α = 0.05, power = 0.80, expected Pearson correlation ρ = 0.95) suggests *n* = 23 participants would be ideal, our repeated-measures design (3 independent 2.5 min segments per participant) yielded 30 paired PPG-ECG measurements per filter-sampling configuration, increasing the effective sample size and precision of CCC estimates. This approach provides adequate statistical power for method comparison studies when the expected concordance is high (CCC ≥ 0.90).

Ten healthy young adults (mean age: 22.3 ± 3.2 years; body mass index: 22.1 ± 2.4 kg/m^2^) were recruited from Hallym University. All participants had normal blood pressure (systolic: 118 ± 8 mmHg, diastolic: 76 ± 6 mmHg), regular sinus rhythm, and no known history of cardiovascular disease, autonomic dysfunction, or arrhythmias. Exclusion criteria included current medication use (including beta-blockers, antiarrhythmics, or psychotropic drugs), smoking, and caffeine consumption within one hour prior to measurement.

This study focused on validating signal processing algorithms rather than investigating physiological characteristics across diverse populations. Therefore, a homogeneous sample of healthy young adults was deliberately chosen to isolate the effects of filter type and sampling frequency while minimizing confounding variables related to age, disease state, or medication. This approach is consistent with methodological recommendations for algorithm validation studies, where population homogeneity enhances internal validity and allows for clearer attribution of observed differences to the technical factors under investigation [28,29].

We acknowledge that this homogeneous sample limits generalizability to broader populations, including elderly individuals, patients with cardiovascular disease, or those with arrhythmias, where PPG signal quality may differ substantially. Future validation studies should include larger, stratified cohorts across age groups, disease states, and demographic characteristics to establish external validity and clinical applicability.

### 2.6. Data Collection Protocol

The PPG sensor was positioned on the index finger of either hand, and ECG electrodes were positioned in a standard Lead II configuration after skin preparation with alcohol. PPG signals were acquired using a custom-built system featuring a 16-bit microcontroller (MSP430F6638, Texas Instruments, Dallas, TX, USA), a 940 nm infrared LED driven at 20 mA, and a silicon PIN photodiode. The signals were digitized by a 12-bit ADC at sampling rates of 1000, 500, and 250 Hz. This device implemented both the Butterworth and Elliptic filters under each sampling condition. The reference ECG signal was acquired simultaneously with the PPG signal using a clinical-grade 3-lead AD8232 ECG module (Analog Devices, Norwood, MA, USA) in a standard Lead II configuration. The signal was sampled at 1000 Hz with 12-bit resolution (using the same ADC as the PPG system). Before R-peak detection, the raw ECG underwent standard preprocessing: a 4th-order Butterworth bandpass filter (0.5–40 Hz) removed baseline wander and high-frequency noise, and a 60 Hz notch filter eliminated powerline interference.

Simultaneous ECG and PPG recordings were acquired in 2.5 min segments under each of the six digital filter-sampling configurations, comprising a combination of two IIR filter types (Butterworth and Elliptic) and three sampling frequencies (1000, 500, and 250 Hz). All six configurations were applied sequentially within a single experimental session. This full sequence was then repeated two additional times, resulting in three independent 2.5 min HRV segments per configuration for each participant. Accordingly, a total of 30 HRV index measurements were obtained per configuration across all 10 participants (3 segments × 10 participants), enabling statistically reliable inter-configuration comparisons.

### 2.7. HRV Metrics and Statistical Analysis

HRV frequency-domain indices were calculated from 2.5 min tachogram segments. The non-equidistant NN interval series was converted to a uniformly sampled representation by cubic spline interpolation and subsequent resampling at 4 Hz [1], producing 600 samples per recording. Spectral power estimation was performed using Welch’s modified periodogram method. The resampled data were segmented into 256-sample epochs with 128-sample overlap (50%). A Hanning window function was applied to each epoch to reduce spectral leakage, followed by 1024-point fast Fourier transformation. The resulting power spectra were averaged across all epochs to obtain the final power spectral density.

The analysis covered the very-low-frequency VLF (0.0033 to 0.04 Hz), low-frequency LF (0.04 to 0.15 Hz), and high-frequency HF (0.15 to 0.40 Hz) bands. To ensure comparability with established ECG-based HRV standards [1], all spectral metrics were log-transformed (ln VLF, ln LF, ln HF), and the sympathetic–parasympathetic balance was calculated as the ratio R = ln(LF/HF). Detailed definitions of all frequency-domain HRV parameters are provided in Table A1 (Appendix A).

Agreement between PPG-derived and ECG-derived HRV indices was evaluated using three statistical methods. Lin’s concordance correlation coefficient (CCC) was used to investigate both precision and accuracy [16,29]. The two one-sided test (TOST) procedure was performed to formally evaluate statistical equivalence within predefined equivalence margins [17]. Finally, Passing–Bablok regression (PBR) was employed to identify any systematic proportional or constant bias [17]. All statistical analyses were carried out using Python 3.10.9 with scipy 1.10.0, with α = 0.05 (significance level).

## 3. Results

### 3.1. Agreement and Statistical Equivalence Analysis of HRV Indices (CCC + TOST)

Paired PPG–ECG recordings were analyzed across six filter–sampling configurations (Butterworth or Elliptic at 1000, 500, and 250 Hz). Agreement for log-transformed HRV indices (ln HF, ln LF, ln VLF) was evaluated using Lin’s concordance correlation coefficient (CCC), which jointly assesses both accuracy and precision [16], and statistically validated using two one-sided equivalence testing (TOST) with ±5% log-scale bounds [17].

Butterworth filters at 1000 Hz and 500 Hz consistently demonstrated excellent concordance (CCC ≥ 0.95) and successfully met the equivalence criterion, with TOST-adjusted *p*-values below the Holm-corrected significance threshold. In contrast, down-sampling to 250 Hz—particularly with Elliptic filters—resulted in noticeably reduced CCC values and a failure to establish statistical equivalence. Among all configurations, Butterworth at 1000 Hz showed the highest overall agreement and equivalence, followed closely by the 500 Hz configuration, whereas Elliptic filters achieved acceptable CCC-based agreement only at 1000 Hz but did not retain equivalence at lower sampling rates.

These findings were consistent across ln HF, ln LF, and ln VLF, confirming that Butterworth filters at ≥500 Hz preserved the highest fidelity to ECG reference measurements, while Elliptic designs became increasingly unstable under spectral compression. Table 3 presents the integrated CCC and TOST results, clearly highlighting the best-performing equivalence-passing condition for each HRV frequency band.

### 3.2. Agreement Statistics by Filter Types and Sampling Frequencies

**Figure 3:**
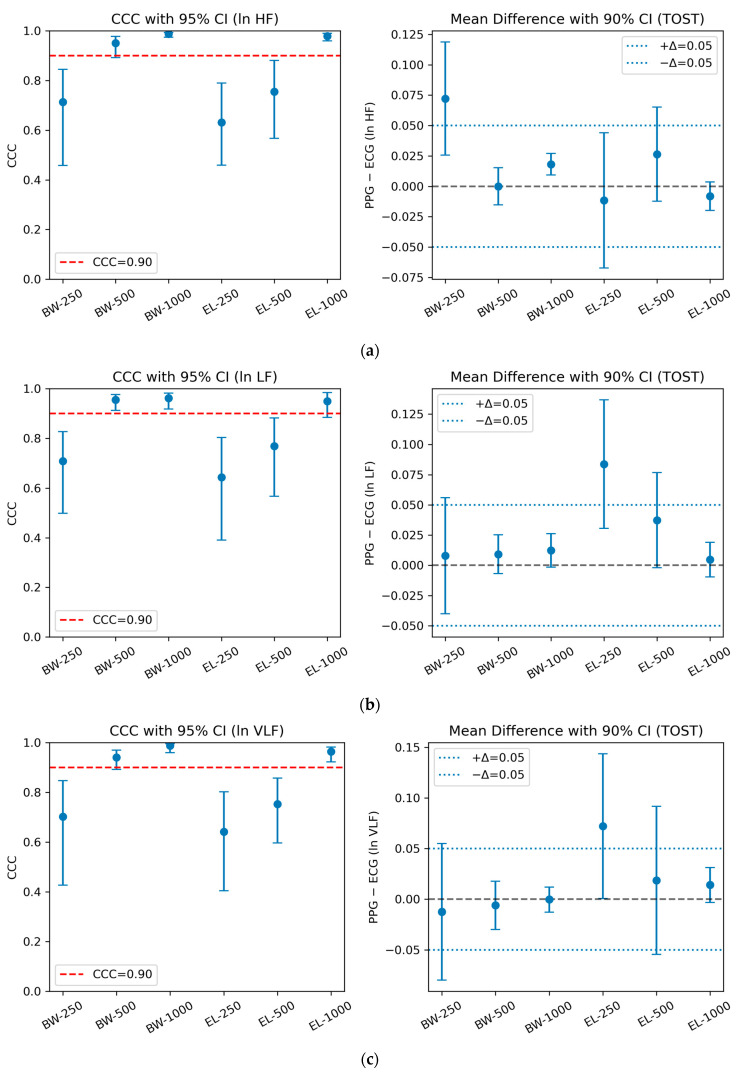
Agreement between PPG- and ECG-derived HRV indices across six filter–sampling configurations. The left panels show Lin’s concordance correlation coefficients (CCC) with 95% bootstrap confidence intervals, with the red dashed line indicating the 0.90 threshold for excellent agreement. The right panels present mean differences with 90% confidence intervals relative to the ±0.05 equivalence bounds (blue dotted lines) used in the TOST procedure. Results are shown for (**a**) ln HF, (**b**) ln LF, and (**c**) ln VLF, comparing Butterworth (BW) and Elliptic (EL) filters at sampling rates of 250, 500, and 1000 Hz.

Consistent with the integrated CCC and TOST analysis, visual inspection of the agreement plots further underscores the superiority of Butterworth filters at higher sampling rates. As shown in Figure 3, agreement trends were evaluated across three HRV bands (ln HF, ln LF, ln VLF), each reported using CCC with 95% confidence intervals (left panels) and mean differences with 90% confidence intervals relative to the ±0.05 equivalence bounds (right panels). The horizontal red line in the CCC panels marks the 0.90 threshold for excellent agreement, while the blue dashed lines in the TOST panels denote the equivalence margins adopted in this study.

### 3.3. Agreement Analysis of PPG and ECG for HRV Frequency Bands Using Bland–Altman Plots

Bland–Altman plots [30] were utilized to evaluate the agreement between PPG- and ECG-derived HRV indices (ln HF, ln LF, ln VLF) across six filter–sampling configurations (Butterworth or Elliptic at 1000, 500, and 250 Hz). Each plot displays 95% limits of agreement (LoA) and the mean difference, enabling visualization of systematic deviation and random variability between PPG and ECG measurements.

**Figure 4:**
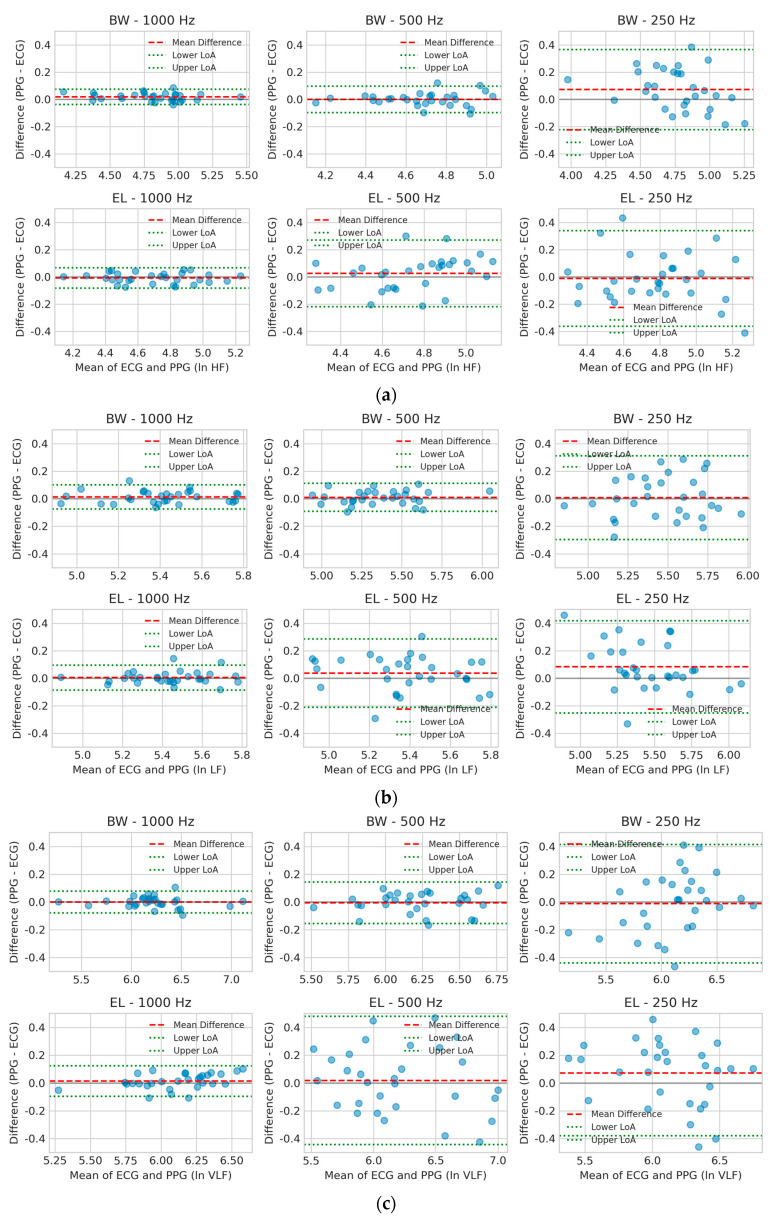
Bland–Altman plots for log-transformed HRV indices comparing PPG- and ECG-derived measurements under six filter–sampling configurations. Results are shown for (**a**) ln HF, (**b**) ln LF, and (**c**) ln VLF. Each panel contains six subplots corresponding to Butterworth (BW) and Elliptic (EL) filters at sampling rates of 1000, 500, and 250 Hz. The x-axis displays the average of PPG and ECG values, and the y-axis plots the difference (PPG − ECG). Red dashed lines indicate the mean bias, while green dotted lines denote the 95% LoA (mean difference ± 1.96 SD), and blue dots represent individual measurements. These plots visualize systematic bias and random variability across filter designs and sampling frequencies.

For ln HF (Figure 4a), Butterworth at 500 Hz and 1000 Hz showed minimal bias and narrow LoA, indicating strong interchangeability with ECG. Elliptic at 1000 Hz also performed acceptably, although with slightly wider dispersion. At 250 Hz, both filters exhibited markedly larger variability, with Elliptic showing the poorest agreement, likely due to ripple-induced phase distortion and aliasing effects.

For ln LF (Figure 4b), Butterworth again outperformed Elliptic, maintaining low bias and tight LoA at 500 Hz and 1000 Hz. In contrast, Elliptic filters—particularly at 250 Hz—displayed greater dispersion and a clear upward bias (PPG > ECG), suggesting susceptibility to low-frequency drift and external noise contamination.

For ln VLF (Figure 4c), variability was inherently higher across all conditions. Butterworth at 1000 Hz showed the smallest bias and narrowest LoA, with 500 Hz remaining acceptable. Elliptic filters—especially at 250 Hz—demonstrated wide LoA and systematic overestimation, indicating poor robustness in very-low-frequency analysis.

Overall, Bland–Altman analysis supports the previous CCC and TOST findings: Butterworth filtering at ≥500 Hz consistently provides the most reliable agreement with ECG, whereas Elliptic filters—most notably at 250 Hz—exhibit reduced stability and increased bias.

### 3.4. Agreement Analysis Using Passing-Bablok Regression

**Figure 5:**
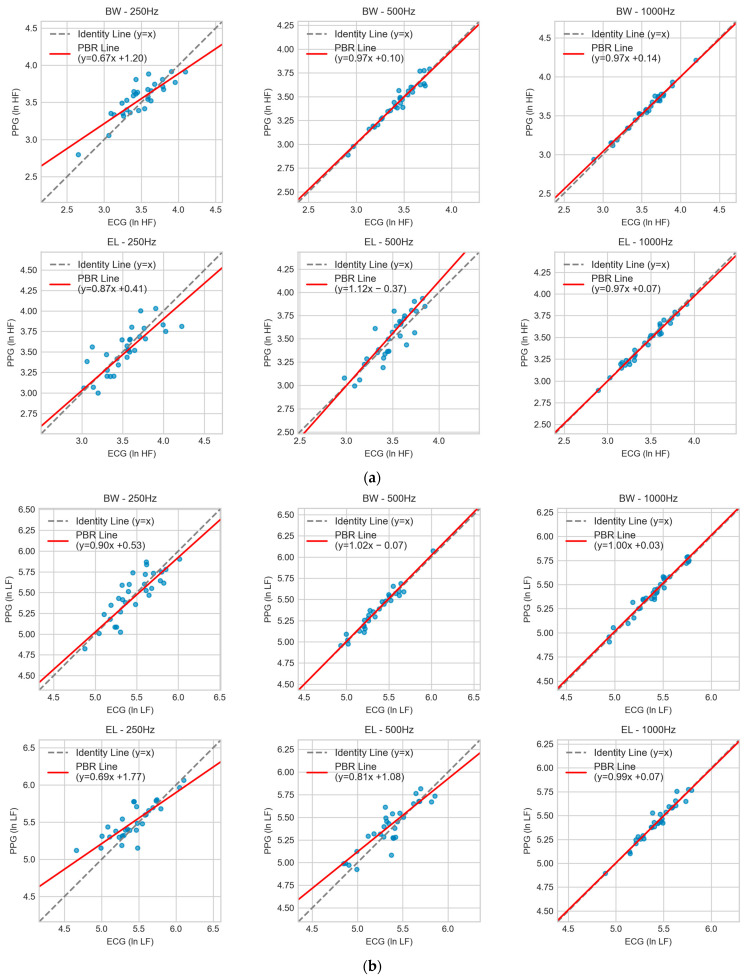

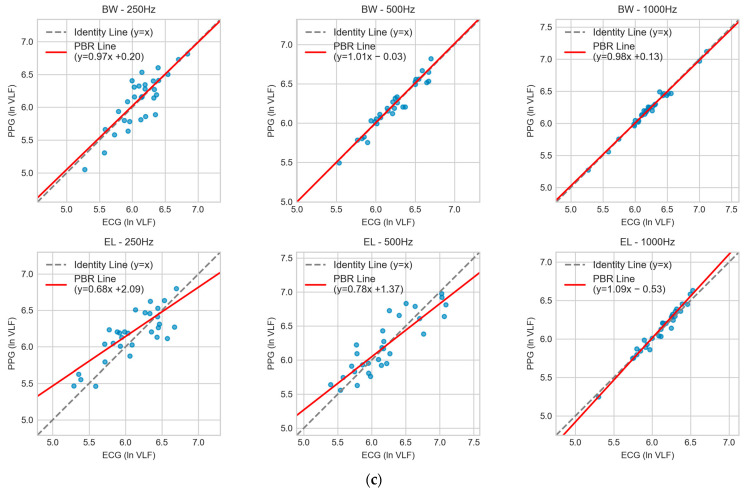
Passing–Bablok regression comparing PPG- and ECG-derived HRV indices across filter–sampling conditions: (**a**) ln HF, (**b**) ln LF, and (**c**) ln VLF. Results are shown for Butterworth (BW) and Elliptic (EL) filters at 1000, 500, and 250 Hz. Scatter points represent paired measurements; the dashed line denotes the line of identity (y = x), and the red line indicates the PBR fit with slope and intercept. Blue dots represent individual HRV indices. Agreement is strongest for BW at 500 and 1000 Hz, acceptable for EL at 1000 Hz, and weakest at 250 Hz, especially for EL.

Passing–Bablok regression (PBR) [17] was applied to evaluate proportional and constant bias between PPG- and ECG-derived HRV indices across the six filter–sampling conditions. Slopes approaching unity and intercepts near zero were interpreted as evidence of minimal systematic error. For ln HF (Figure 5a), Butterworth filters at 500 Hz and 1000 Hz produced regression lines close to identity (slopes ≈ 0.97 with small intercepts of +0.10 and +0.14, respectively). At 250 Hz, Butterworth performance deteriorated (slope 0.67, intercept +1.20), reflecting marked proportional error and positive bias. Elliptic filtering at 1000 Hz (0.97x + 0.07) yielded acceptable results, but deviations became evident at 500 Hz (1.12x − 0.37) and 250 Hz (0.87x + 0.41), where systematic errors increased with lower sampling rates. For ln LF (Figure 5b), Butterworth again demonstrated reliable agreement at 500 Hz (1.02x − 0.07) and 1000 Hz (1.00x + 0.03). At 250 Hz, however, the slope dropped to 0.90 with an intercept of +0.53, indicating proportional underestimation and bias. Elliptic at 1000 Hz (0.99x + 0.07) was comparable to Butterworth, but 500 Hz (0.81x + 1.08) and 250 Hz (0.69x + 1.77) showed pronounced bias and poor reproducibility. For ln VLF (Figure 5c), variability was more pronounced across all conditions. Butterworth at 500 Hz (1.01x − 0.03) and 1000 Hz (0.98x + 0.13) maintained slopes close to unity with negligible intercepts, whereas at 250 Hz (0.97x + 0.20) a constant bias emerged. Elliptic filters showed stronger deviations, particularly at 250 Hz (0.68x + 2.09) and 500 Hz (0.78x + 1.37), with the 1000 Hz condition (1.09x − 0.53) also showing notable deviation. In these cases, the regression fits diverged substantially from the line of identity.

Taken together, the regression analysis confirmed that Butterworth filtering at ≥500 Hz minimized both proportional and constant bias, consistent with the outcomes from CCC and Bland–Altman analyses. Conversely, Elliptic filters—especially at 250 Hz—demonstrated significant systematic error, limiting their interchangeability with ECG-derived indices.

### 3.5. Spectral Fidelity Analysis

Spectral fidelity was assessed by calculating the similarity between the frequency response and an ideal bandpass filter for each filter condition. The spectral fidelity metric quantifies how closely the implemented filter approximates the ideal bandpass characteristics across the frequency range of interest. Since zero-phase forward-backward filtering was applied in this study, the filter is applied twice. This results in the magnitude response being squared, effectively doubling the filter order from 2nd-order to 4th-order equivalent. The spectral fidelity (SF) was computed using the following equation [8]:

(7) $$SF=\left(1-\frac{\sum_{k=1}^{K}{\left(\left|H_{ideal}\left(f_{k}\right)\right|-\left|H_{actual}\left(f_{k}\right)\right|\right)}^{2}}{\sum_{k=1}^{K}{\left|H_{ideal}\left(f_{k}\right)\right|}^{2}}\right)\times 100\%$$

where $H_{ideal}\left(f_{k}\right)$ represents the amplitude response of an ideal bandpass filter at frequency $f_{k}$, ${\left|H_{actual}\left(f_{k}\right)\right|}^{2}$ is the magnitude spectrum of the implemented filter at the same frequency, and K is the total number of frequency points evaluated across the spectrum. The ideal bandpass filter is defined as having unity gain (0 dB) within the passband (0.8–20 Hz) and complete attenuation outside this range. This metric provides a comprehensive measure of filter performance by accounting for deviations in both the passband and stopband regions.

**Figure 6:**
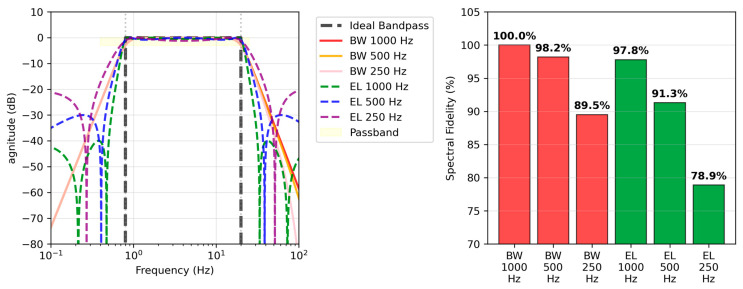
Frequency response characteristics and spectral fidelity comparison for each filter condition. **Left**: Comparison of frequency responses with ideal bandpass filter (black dashed line), showing that Butterworth filters exhibit flat characteristics in the passband (0.8–20 Hz, yellow region) while elliptic filters demonstrate sharp cutoff characteristics with passband ripple. **Right**: Spectral fidelity relative to ideal bandpass filter, where BW filters (1000 Hz: 100.0%, 500 Hz: 98.2%, 250 Hz: 89.5%) generally outperform EL filters (1000 Hz: 97.8%, 500 Hz: 91.3%, 250 Hz: 78.9%), with BW 500 Hz particularly providing high fidelity while maintaining computational efficiency.

The results showed that the Butterworth filter generally exhibited superior fidelity compared to the Elliptic filter (Table 4 and Figure 6). Notably, the BW 500Hz filter achieved a high spectral fidelity of 98.2%, offering approximately 50% reduction in computational load with only a 1.8% performance degradation compared to the BW 1000 Hz (100.0%). The 500 Hz Butterworth filter provides an optimal balance between accuracy and efficiency, making it the most practical choice for wearable devices where both battery life and measurement accuracy are important.

## 4. Discussion

This pilot study systematically compared Butterworth and Elliptic IIR filters at three sampling frequencies (1000, 500, 250 Hz) to identify the optimal preprocessing configuration for PPG-based HRV analysis in wearable devices. The controlled laboratory setting with homogeneous participants (*n* = 10 healthy young adults) was deliberately chosen to isolate the effects of filter type and sampling frequency while minimizing confounding variables. Although this design limits immediate clinical generalizability, it provides a methodologically rigorous foundation for identifying optimal configurations worthy of large-scale validation.

Our principal finding is that Butterworth filtering at 500 Hz emerged as the optimal configuration. It achieved excellent concordance with ECG-derived HRV, as evidenced by a Concordance Correlation Coefficient (CCC) of ≥ 0.94, successful TOST equivalence testing within a ±5% margin, and Passing-Bablok regression slopes between 0.97 and 1.02. This configuration also preserved 98.2% of the signal’s spectral fidelity while halving the computational load compared to 1000 Hz. While the 1000 Hz Butterworth filter offered marginally stronger agreement (CCC ≈ 0.96–0.99), the minimal accuracy gain does not justify the doubled computational cost for battery-constrained wearables. In contrast, Elliptic filters consistently underperformed due to passband ripple and nonlinear phase response, which introduced systematic bias, particularly at 250 Hz (CCC ≈ 0.64). The poor performance at 250 Hz for Butterworth filter (CCC ≈ 0.71) underscores the risks of excessive downsampling and provides quantitative evidence against the common industry practice of using this frequency in low-cost wearables.

The present study focused exclusively on IIR filters (Butterworth and Elliptic), motivated by the computational and memory constraints of wearable devices. IIR filters achieve steep roll-off with significantly fewer coefficients than equivalent FIR designs, making them well-suited for battery-powered applications. The comparison between Butterworth (maximally flat passband) and Elliptic (maximal selectivity with ripple) represents a fundamental trade-off in IIR filter theory, and prior studies have demonstrated Butterworth’s superiority for cardiovascular signal processing due to phase linearity and absence of passband ripple [8,21,22]. We acknowledge that other filter types—including Chebyshev (steeper roll-off with ripple), Bessel (linear group delay), and linear-phase FIR designs—offer alternative characteristics that may be advantageous in specific contexts. Bessel filters may further improve peak timing accuracy, while FIR filters eliminate phase distortion without forward-backward processing, albeit at higher computational cost. A comprehensive comparison including these designs would provide valuable insights and represent an important direction for future research.

These results extend our previous work [14], which found Butterworth filters superior for NN interval extraction but lacked systematic frequency evaluation and direct ECG validation. The present study makes three key advances: (1) simultaneous PPG-ECG recordings for validation against the clinical gold standard, (2) a systematic comparison of sampling frequencies, and (3) comprehensive statistical validation using four complementary methods. Our recommendation of a ≥500 Hz sampling rate appears higher than the 20–50 Hz suggested by Béres et al. [13], a difference attributable to our focus on frequency-domain parameters (ln HF, ln LF, ln VLF) without interpolation, where spectral fidelity is paramount. Filter-induced distortions at lower sampling rates and our stricter accuracy criteria (CCC ≥ 0.94, TOST equivalence) further justify the need for higher sampling rates to maintain clinical-grade accuracy in real-time, frequency-domain HRV analysis.

From a clinical perspective, the statistical equivalence demonstrated between HRV parameters derived from PPG with a 500 Hz Butterworth filter and those from the reference ECG is highly significant [31,32]. This high degree of agreement suggests that wearable devices implementing this configuration could serve as a reliable tool for applications such as detecting autonomic dysfunction, tracking stress recovery, and remotely monitoring cardiovascular patients, yielding results that are statistically comparable to traditional ECG-based assessments under specified conditions [1]. Validation is essential for integrating wearable-generated data more broadly into electronic health records and for informing clinical decision support systems (CDSS), addressing key challenges in system interoperability and data privacy [33]. Furthermore, by demonstrating equivalence with established reference methods through fit-for-purpose validation, these findings may help streamline the regulatory approval process for PPG-based medical devices [34].

From an engineering standpoint, the 50% reduction in computational load achieved by decreasing the sampling frequency from 1000 Hz to 500 Hz yields significant advantages in resource efficiency. This reduction can extend device battery life by an estimated 15–20%—equivalent to an additional 8–12 h for a typical 200 mAh battery—under specific operating conditions [35]. Moreover, it enables the selection of lower-power analog-to-digital converters (ADCs), such as a 12-bit SAR ADC instead of a more power-intensive 16-bit delta-sigma variant and simplifies the design of the prerequisite anti-aliasing filter [36,37]. The corresponding halving of memory requirements, from 300,000 to 150,000 samples for a standard five-minute analysis window, further alleviates hardware constraints. These factors define a clear engineering trade-off, allowing developers of microcontroller-based wearables to balance signal fidelity against hardware limitations, thereby accelerating the development and deployment of next-generation, clinical-grade HRV monitoring solutions.

Finally, these findings have important implications for machine learning (ML) applications in wearable health. Robust preprocessing is essential, as inaccuracies can introduce systematic biases that propagate through AI pipelines and degrade model performance in areas like stress detection, sleep staging, and cardiovascular risk assessment [38,39]. By providing reproducible benchmarks for accuracy and spectral fidelity, this study offers reference criteria for validating preprocessing pipelines before ML integration. Adopting a standardized preprocessing approach, such as the 500 Hz Butterworth filter, also facilitates direct comparison of ML model performance across studies and minimizes distribution shift when models trained on ECG-derived HRV are deployed on PPG-based devices.

### 4.1. Study Limitations

This work should be viewed as a preliminary feasibility study rather than definitive clinical evidence, as the sample was limited to 10 healthy young adults, limiting statistical power and generalizability. Filter performance may differ in elderly individuals and patients with arrhythmias, heart failure, or autonomic neuropathy, where PPG signal quality is often degraded; thus, future studies should include larger and stratified cohorts by age, sex, ethnicity, and cardiovascular disease status. All measurements were conducted under controlled laboratory resting conditions, whereas real-world wearable environments involve motion artifacts, variable skin contact, ambient light interference, and diverse physiological states (e.g., exercise, sleep, stress), requiring multi-center ambulatory validation over days to weeks to confirm the robustness of the Butterworth 500 Hz configuration. The analysis focused on HRV frequency-domain metrics (ln HF, ln LF, ln VLF), which are most sensitive to filter characteristics, but HRV time-domain indices (SDNN, RMSSD, pNN50) and nonlinear metrics (sample entropy, DFA) may exhibit different responses and warrant comprehensive future evaluation.

An important limitation of the present study is the lack of a real-time implementation analysis. The zero-phase forward-backward filtering employed here is inherently non-causal, requiring access to the entire signal segment and thus introducing a latency proportional to the filter order and segment length. For the 2.5 min segments analyzed, this approach is suitable for offline or batch processing but not for strict real-time applications requiring instantaneous output.

For a real-time wearable implementation, several considerations arise: (1) Computational delay: A 4th-order IIR filter requires approximately 9 arithmetic operations (5 multiplications and 4 additions) per sample, resulting in approximately 4500 operations per second at 500 Hz sampling—well within the capability of modern 16-bit microcontrollers. (2) Memory requirements: Buffering a 30 s sliding window at 500 Hz requires only 15 KB for 16-bit samples, which is feasible for low-power devices. (3) Causal filtering alternative: For real-time applications, a single-pass (causal) Butterworth filter could be employed, accepting the trade-off of phase distortion, or the group delay could be compensated for algorithmically.

Future work should evaluate causal filter implementations and quantify the trade-off between real-time responsiveness and HRV accuracy. Hybrid approaches, such as applying zero-phase filtering to short, overlapping windows with appropriate windowing functions, may offer a practical compromise for near-real-time applications.

Given the exploratory nature of this work, we reported uncorrected *p*-values across 18 comparisons (3 bands × 6 configurations) to avoid excessive Type II error, consistent with recommendations for hypothesis-generating studies [40]. Nevertheless, this pilot makes a meaningful contribution by providing the first systematic comparison of filter types across sampling frequencies using simultaneous PPG-ECG recordings and four statistical validation methods, delivering immediately actionable engineering insights (98.2% spectral fidelity and ~50% computational load reduction), reproducible statistical benchmarks (CCC ≥ 0.94 and slopes 0.97–1.02), and evidence-based guidelines that address a critical gap in the literature regarding filter–sampling interaction effects for optimized PPG-based HRV preprocessing.

### 4.2. Future Work

Future research should prioritize large-scale multicenter validation across diverse populations, clinical conditions, and real-world monitoring scenarios to establish generalizability and clinical utility. Future work should also expand beyond frequency-domain HRV outcomes to establish filter impact profiles across time-domain, nonlinear, and ultra–short-term HRV indices, enabling application-specific optimization (e.g., mental stress detection vs. cardiac risk pre-screening). Building upon the present static-filter baseline, adaptive signal processing frameworks that dynamically adjust bandwidth or sampling rate in response to evolving signal quality may offer a pathway toward intelligent energy–accuracy tradeoff management in resource-constrained wearables. Finally, integrating validated preprocessing pipelines with predictive machine learning models and multi-sensor fusion architecture (e.g., combining PPG-HRV with electrodermal activity, skin temperature, or motion context) is expected to advance personalized digital biomarkers and proactive intervention systems.

## 5. Conclusions

This study systematically evaluated filter types and sampling frequencies for PPG-based HRV analysis, establishing Butterworth filtering at ≥500 Hz as the optimal preprocessing configuration. This approach achieved statistical equivalence with ECG-derived HRV parameters while reducing computational load by 50% compared to 1000 Hz sampling, demonstrating that clinical-grade accuracy and engineering feasibility are not mutually exclusive.

The validated preprocessing standard provides three key contributions: (1) evidence-based guidelines for wearable device developers to balance signal fidelity with hardware constraints, (2) a robust foundation for machine learning applications where accurate preprocessing prevents systematic bias, and (3) reproducible benchmarks that facilitate direct comparison across studies and accelerate regulatory approval pathways.

By bridging clinical accuracy and engineering feasibility, these findings support the translation of PPG-based HRV monitoring into clinically adopted, consumer-accessible wearable health technologies. Future validation in diverse populations and real-world conditions will be essential to confirm generalizability and support widespread clinical adoption.

## Figures and Tables

**Table 1:** Filter coefficients for the 2nd-order prototype (0.8–20 Hz) across sampling frequencies (*f_s_*), producing a 4th-order equivalent response after zero-phase forward-backward processing.

Coefficient	Numerator B[z]			Denominator A[z]		
	1000 Hz	500 Hz	250 Hz	1000 Hz	500 Hz	250 Hz
Butterworth Filter						
b_0_/a_0_	0.00334894	0.00308602	0.01145082	1.00000000	1.00000000	1.00000000
b_1_/a_1_	0.00000000	0.00000000	0.00000000	−3.82854718	−3.83439915	−3.66597653
b_2_/a_2_	−0.00669788	−0.00617203	−0.02290164	5.50034780	5.51815832	5.05449352
b_3_/a_3_	0.00000000	0.00000000	0.00000000	−3.51495413	−3.53292690	−3.10959869
b_4_/a_4_	0.00334894	0.00308602	0.01145082	0.84315388	0.84916874	0.72110356
Elliptic Filter						
b_0_/a_0_	0.00344194	0.00317162	0.01176538	1.00000000	1.00000000	1.00000000
b_1_/a_1_	−0.00037360	−0.00034423	−0.00127665	−3.85987946	−3.86728685	−3.70054878
b_2_/a_2_	0.00613666	0.00565358	0.02096706	5.59596629	5.61608290	5.13616839
b_3_/a_3_	−0.00037360	−0.00034423	−0.00127665	−3.61221511	−3.63471464	−3.19083711
b_4_/a_4_	0.00344194	0.00317162	0.01176538	0.87612866	0.88570859	0.75545935

**Table 2:** Frequency–response parameters for Butterworth (BW) and Elliptic (EL) bandpass filters (0.8–20 Hz) at multiple sampling frequencies. Passband ripple (rp); low-frequency transition width ($f_{low\_tw})$ high-frequency transition width ($f_{high\_tw}$); group-delay spread in samples at fs ($\tau_{g}\left(\omega \right)$); and phase variation in degrees (Phase).

Filter	fs (Hz)	f_low_tw_ (Hz)	f_high_tw_ (Hz)	rp (dB)	$\tau_{g}\left(\omega \right)$	Phase
BW	1000	0.65	84.35	0.0	13–300	±90
	500	0.65	75.36	0.0	6.5–150	±90
	250	0.65	55.34	0.0	3.25–75	±90
EL	1000	0.65	82.80	1.0	12–290	±80
	500	0.65	74.18	1.0	6–145	±80
	250	0.65	54.74	1.0	3–72	±80

**Table 3:** Integrated agreement and equivalence analysis between PPG- and ECG-derived HRV indices (ln HF, ln LF, ln VLF). Lin’s CCC (95% bootstrap CI), bias correction factor ($C_{b}$), Pearson’s correlation (r), mean difference with 90% CI, and Holm-adjusted TOST results (±5% log-scale bounds) are jointly presented based on paired data (*n* = 30 per filter-sampling condition).

HRV	Filter	$f_{s}$(Hz)	CCC (95% CI)	r	$C_{b}$	Mean diff (90% CI)	Equiv (Holm)	p_TOST_adj
ln HF	BW	250	0.713 (0.458, 0.845)	0.866	0.823	0.072 (0.026, 0.119)	No	0.788
	BW	500	0.950 (0.892, 0.978)	0.975	0.975	0.000 (−0.015, 0.015)	Yes	0.000
	BW	1000	0.988 (0.974, 0.993)	0.995	0.993	0.018 (0.009, 0.027)	Yes	0.000
	EL	250	0.631 (0.459, 0.790)	0.796	0.793	−0.012 (−0.067, 0.044)	No	0.375
	EL	500	0.755 (0.567, 0.880)	0.879	0.860	0.026 (−0.012, 0.065)	No	0.375
	EL	1000	0.979 (0.959, 0.989)	0.990	0.989	−0.008 (−0.020, 0.004)	Yes	0.000
ln LF	BW	250	0.709 (0.499, 0.827)	0.843	0.841	0.008 (−0.040, 0.056)	No	0.239
	BW	500	0.955 (0.912, 0.976)	0.978	0.977	0.009 (−0.007, 0.025)	Yes	0.000
	BW	1000	0.961 (0.918, 0.982)	0.981	0.980	0.012 (−0.002, 0.026)	Yes	0.000
	EL	250	0.643 (0.390, 0.803)	0.830	0.775	0.084 (0.031, 0.137)	No	0.863
	EL	500	0.768 (0.567, 0.882)	0.882	0.871	0.037 (−0.002, 0.077)	No	0.627
	EL	1000	0.950 (0.884, 0.984)	0.975	0.974	0.005 (−0.010, 0.019)	Yes	0.000
ln VLF	BW	250	0.702 (0.426, 0.847)	0.847	0.829	−0.012 (−0.080, 0.055)	No	0.526
	BW	500	0.940 (0.892, 0.969)	0.970	0.969	−0.006 (−0.030, 0.018)	Yes	0.007
	BW	1000	0.987 (0.960, 0.995)	0.994	0.994	−0.000 (−0.013, 0.012)	Yes	0.000
	EL	250	0.641 (0.404, 0.802)	0.813	0.788	0.072 (0.001, 0.143)	No	0.698
	EL	500	0.752 (0.597, 0.858)	0.870	0.865	0.019 (−0.054, 0.092)	No	0.526
	EL	1000	0.964 (0.922, 0.982)	0.984	0.979	0.014 (−0.003, 0.031)	Yes	0.003

**Table 4:** Spectral Fidelity (SF) and Performance Metrics.

Filter fs	SF (%)	Passband Flatness	Stopband Performance	Phase Linearity	Overall Score
BW 1000 Hz	100.0	Excellent (0.05 dB)	Good	Excellent	95
BW 500 Hz	98.2	Excellent (0.06 dB)	Good	Excellent	97
BW 250 Hz	89.5	Good (0.12 dB)	Moderate	Good	78
EL 1000 Hz	97.8	Moderate (1.0 dB)	Excellent	Moderate	88
EL 500 Hz	91.3	Moderate (1.0 dB)	Excellent	Moderate	82
EL 250 Hz	78.9	Poor (1.2 dB)	Good	Poor	68

## Data Availability

Data supporting the findings of this study are available from the corresponding author upon request. Public availability is restricted due to privacy concerns.

## Abbreviations

The following abbreviations are used in this manuscript:

BW	Butterworth filter
EL	Elliptic filter
LoA	Limits of agreement
HRV	Heart rate variability
TP	Total power
VLF	Very low frequency
LF	Low frequency
HF	High frequency
PPG	Photoplethysmography
fs	Sampling frequency
IIR	Infinite impulse response
SF	Spectral fidelity

## Appendix A

Table A1 summarizes the spectral HRV indices employed in this study. The parameter ln TP (total power) represents the power spectral density (PSD) integrated over the entire frequency range, whereas ln VLF corresponds to the PSD in the very low-frequency band (0.0033 to 0.04 Hz). The parameter *ln LF* denotes the PSD in the low-frequency band (0.03 to 0.15 Hz), and ln HF indicates the PSD in the high-frequency band (0.15 to 0.40 Hz). Finally, the R ratio is defined as the logarithmic ratio of low-frequency to high-frequency power (ln LF/ln HF).

**Table A1:** Frequency-domain HRV Parameters.

Parameter	Unit	Description
ln TP (total power)	ms^2^	PSD in all the frequency bands
ln VLF	ms^2^	PSD in very-low-frequency band (0.0033 to 0.04Hz)
ln LF	ms^2^	PSD in low-frequency band (0.03 to 0.15Hz)
ln HF	ms^2^	PSD in high-frequency band (0.15 to 0.4Hz)
R ratio	-	ln LF/ln HF
